# ATG5 and ATG7 induced autophagy interplays with UPR via PERK signaling

**DOI:** 10.1186/s12964-019-0353-3

**Published:** 2019-05-06

**Authors:** Wei Zheng, Weiwei Xie, Danyang Yin, Rui Luo, Min Liu, Fengjin Guo

**Affiliations:** Department of Cell Biology and Genetics, Core Facility of Development Biology, Chongqing Medical University, Chongqing, 400016 China

**Keywords:** ATG5, ATG7, Autophagy, ER stress, ER-phagy, Apoptosis

## Abstract

**Background:**

Autophagy and ER stress are involved in maintaining some well-orchestrated mechanisms aimed at either restoring cellular homeostasis or performing cell death. Autophagy is a well-defined process which governs overall cellular stress outcomes. Selective degradation of the ER mediated by autophagy occurs through a specific type of autophagy called ER-phagy, which ensures ER protein homeostasis.

**Methods:**

Immunoblotting and RT-PCR were used to evaluate the expression of ATG5 and ATG7 in chondrocyte. Western blotting, Flow cytometry,immunofluorescence cell staining and confocal microscope were used to examine the effect of ATG5 and ATG7 on autophagy, ER stress, cell apoptosis and cell proliferation. Transmission electron microscope and confocal microscope were performed to visualize the autophagy flux and autolysosome formation. The role of ATG5 and ATG7 overexpression on the PERK pathway inhibitor were detected by immunoblotting and treatment with inhibitors.

**Results:**

In current study, we demonstrated that Tm-induced ER stress can activate autophagy while Rapamycin-induced autophagy can inhibit ER stress in chondrocyte. Autophagy related protein ATG5 or ATG7 can promote autophagy and inhibit ER stress individually, and their combined effect can further improve the autophagy enhancement and the ER stress repression. Moreover, ATG5, ATG7 and ATG5 + ATG7 lead cells into more S phase, increase the number of S phase and inhibit apoptosis as well. ATG5, ATG7 and ATG5 + ATG7 regulate autophagy, ER stress, apoptosis and cell cycle through PERK signaling, a vital UPR branch pathway.

**Conclusions:**

ATG5 and ATG7 connect autophagy with ER stress through PERK signaling. The protective effect of ATG5/7 overexpression on chondrocyte survival relys on PERK signaling. The effect of siPERK and siNrf2 on the cytoprotective effect of ATG5/7 are of synergism, while the effect of siPERK and siATF4 are of antagonism. PERK signal may be the pivot for autophagy, ER homeostasis and ER-phagy in chondrocyte.

**Electronic supplementary material:**

The online version of this article (10.1186/s12964-019-0353-3) contains supplementary material, which is available to authorized users.

## Background

The endoplasmic reticulum (ER) is an elaborate cellular organelle essential for cell function and survival. Autophagy, ER stress and apoptosis are closely connected with ER. It’s well known that autophagy in mammalian systems occurs under basal conditions and can be stimulated by stresses like hypoxia, starvation, rapamycin etc. Autophagy can prevent cells from many kinds of stress and was beneficial for cell survival.

In the process of autophagy, the damaged or dysfunctional organelles and macromolecules are encapsulated in the double membrane structure called autophagosome which will then degrade the macromolecule components after fusing with the lysosomes to form autolysosomes to maintain homeostasis of the cells [1–3]. Cell death will happen when autophagy is inhibited, implying autophagy as a cytoprotective mechanism [4, 5]. There are two ubiquitin-like conjugatin systems necessary for the phagophore membrane elongation, including ATG12-ATG5- ATG16L1 autophagosomal precursor formation [6–8] and LC3-I/LC3-II production, which is involved in fusing autophagosome with lysosome to form autolysosomes [9–11]. All is known that autophagy function and morphology are intimately associated to ER, which is necessary for the cell survival under normal condition. The ER stress will be stimulated once beyond the function of the ER [12–14], and the unfolded protein response (UPR) will be activated when some endogenous or exogenous factors influence the homeostasis of ER. ER-phagy exists after selective degradation of the ER by autophagy,and play a key role in the physiology of secretory cells in vivo. ER stress and UPR directly engage and modulate general autophagic flux and direct ER-phagy. Smith et al. identify ER membrane protein CCPG1, as an ER-phagy receptor that interacts with autophagy-related components LC3, GABARAPs and FIP200, maintains ER homeostasis during both physiological and stress conditions [15–17].

Many studies reported that a variety of physical and chemical factors can turn on ER stress and influence cell survival in chondrocyte differentiation, chondrogenesis and endochondral ossification [18–20]. ER stress-induced cell apoptosis will be switched on when stress continues to occur or the cell is unable to accommodate ER stress [21–23]. ER stressors, like tunicamycin, thapsigargin, or DTT, stimulate the autophagosomes formation [24]. The activation of autophagy under ER stress may have a cytoprotective effect and promote cell survival [25–27]. ATG5 and ATG7, as two important autophagy related proteins, increased antophagy and reduced the damaged organelles or degraded macromolecules which accumulated in chondrocytes of cartilage degeneration, then maintained the homeostasis of chondrocyte and were conducive to cell survival [28–30]. However, when and how to modulate autophagy during ER stress is not entirely clear,the direct correlation between these two processes remains unknown.This study aim to clarify the effect of ATG5 and ATG7 on how to regulate ER stress, autophagy and cell survival. Specifically, the data presented herein elucidate the relationship between autophagy, ER stress and ER-phagy. ATG5 and ATG7, as two conventional autophagy-related genes, are involved in ER turnover through PERK signaling. It is of significant interest to clarify the reason behind treatment with autophagy inducer is beneficial to the removel of cytosolic aggregates.

## Methods

### Adenoviruses and plasmids

To generate ATG5 and ATG7 overexpression adenovirus, the cDNA of ATG5 and ATG7 genes were cloned into the pAdTrack-CMV and recombinated to adenovirus according to the instruction (primers: forward, 5′-GTCAGATCCGCTAGAGATCT GCTTACTAAGTTTGGCTTTGGTT-3′ and reverse, 5′-GATATCTTATCTAGAAGC TTAAGGGTGACATGCTCTGATAAAT-3′ for ATG5; forward, 5′-GTC AGATCCGCTAGAGATCTAAATAATGGCGGCAGCTACGG-3′ and reverse, 5′-GATATCTT ATCTAGAAGCTTGGGCCATCTCAGATGGTCTCATC-3′ for ATG7) [30]. Futhermore, the cDNA of ATG7 (forward, 5′-GTTTAAACGGGCCCTCTA GAAAATAATGGCGGCAGCTACGG-3′ and reverse, 5′-TGGAATTCTGCAGATA TCGGGCCATCTCAGATGGTCTCATC-3′) was cloned into pcDNA3.1(−) and pcDNA3.1(−)-ATG5 was presented by Dr. Chuanju Liu of New York University. All of the constructs were verified by endonuclease digestion and nucleic acid sequencing.

### Cell culture

C28I2 cells (a gift from Dr. Chuanju Liu, New York University School of Medicine, New York, NY, USA) were cultivated in DMEM (Gibco, Grand Island, NY, USA) medium supplemented with 10% fetal bovine serum in 10% fetal bovine serum supplemented DMEM (Gibco, Grand Island, NY, USA), and incubated at 37 °C in a humidified atmosphere of 5% CO2. Next, the cells were briefly trypsinized and resuspended into the 60 mm cell culture dish (Biologix, USA), then incubated for 12 h under standard conditions before treatment. To confirm the effects of ATG5, ATG7, siPERK and siNrf2 on chondrocyte. C28I2 cells were infected with Ad-ATG5 (MOI = 80), Ad-ATG7 (MOI = 100), siPERK (50 nmol) or siNrf2 (50 nmol) and Ad-GFP prior to culture 24 h [31, 32]. pcDNA3.1(−)-ATG5 and pcDNA3.1(−)-ATG7 were transfected into cells for 24 h by liposome 8000(Invitrogen) according to the manufacturer’s protocol.

### RNA extraction and reverse transcription (RT)-PCR

RNeasy Mini Kit (BioTeke, Chinese) and PrimeScript RT reagent Kit (TAKARA, USA) were used to extract total RNA and reverse transcription respectively based on the instructions. The specific sequence primers were designed as follows: forward, 5′-AAGCAACTCTGGATGGGATT-3′ and reverse, 5′-GCAGCCACAGGACGA AAC-3′ for ATG5; forward, 5′-CAGTCCGTTGAA GTCCTC-3′ and reverse, 5′-TCAGTGTCCTAGCCACATTAC-3′ for ATG7; forward, 5′-AGGTCGGTG TGAACGGATTTG-3′ and reverse, 5′-GGGGTCGTTGATGGC AACA-3′ for GAPDH. The targeted PCR amplification products were verified by gel-purified bands (Qiagen). The quantitation of RNA was analysed by the BIO-RAD CFX Connect Real-Time PCR system (BIO-RAD). GAPDH was employed as an internal control. And annealing temperatures for these primers were 55 °C except ATG5 which was 52 °C. Data were analyzed by the Relative Quantification (2^-ΔΔCt^) method.

### Western blotting

Total proteins were extracted by RIPA lysis buffer which mixed with proteinase inhibitor (PMSF). The proteins were next separated by 12% SDS-PAGE and transfered to the PVDF membrane (Millipore). Then blocking for 2 h in 5% non-fat dry milk which was dissolved with 1 × Tris-buffered saline and incubated with the corresponding antibody [ATG5 (NB110–53818; NOVUS 1:500); ATG7 (MAB6608; R&D 1:500); LC3 (NB100–2220; NOVUS 1:1000); SQSTM1/p62 (ab56416; abcam 1:1000); Caspase-3 (#9662; CST 1:1000); Caspase-12 (ab62484; abcam 1:1000); PERK (sc-13,073; Santa Cruz 1:200); p-PERK (sc-32,577; Santa Cruz 1:200); Nrf2 (sc-722; Santa Cruz 1:500); XBP1 (sc-7160; Santa Cruz 1:200); ATF4 (#11815; CST 1:1000); IRE1 (NB100–2323; NOVUS 1:1000); β-actin (B1033; Biodragon 1:8000)] at 4 °C overnight. The next day, the HRP-conjugated goat anti-mouse or anti-rabbit IgG was added to incubate for 2 h after washing for thrice. At last, an enhanced chemiluminescence reagent (Beyo ECL Plus, Beyotime) was used to visualized the band.

### Flow cytometry

On basis of references [33, 34], when C28I2 cells were incubated with the adenoviruses or controls for 24 h, the cells were washed, trypsinized and resuspended with 1 × PBS for FCM assay or fixed the cells with 75% alcohol for determing the distribution of cell cycle. These experiments were conducted thrice.

### Immunofluorescence cell staining and confocal microscope

Cells were planted on coverslips and infected with the adenoviruses or transfected with the plasmids for 24 h. Then washing for twice with 1 × PBS and fixed the cells with 4% paraformaldehyde for 30 min. Next permeabilized with Triton X-100 (0.1% in PBS) for 20 min and blocked with 5% BSA for 1 h. Next incubating the cells with LC3 (1:200) and LAMP1 (1:200) at 4 °C overnight. The next day, the cells were incubated with HRP-conjugated goat anti-rabbit IgG (1:300, abbkine) for 1 h darkly at room temperature after washing with 1 × PBS thrice. Then the 4–6-diamidino-2-phenylindole (DAPI) was added for 15 min. Finally, using a confocal microscope (Nikon) to collect the corresponding images.

### Transmission electron microscope

After incubated with the adenoviruses or controls for 24 h, the cells were washed, trypsinized and resuspended.Then fixed the cells with glutaraldehyde for transmission electron microscope analysis to detect autophagosomes. Transmission electron microscope (JEM-1400PLUS) was used to collect the corresponding images.

### Statistical analysis

Date were analyzed by GraphPad Prism 5 software and student’s t-test for single comparisons. These data come from at least 3 independent experiments and expressed as means±SD. Moreover, *P < 0.05, **P < 0.01, ***P < 0.001 were considered statistically significant.

## Results

### ER stress interplays with autophagy in human chondrocyte

**Fig. 1:**
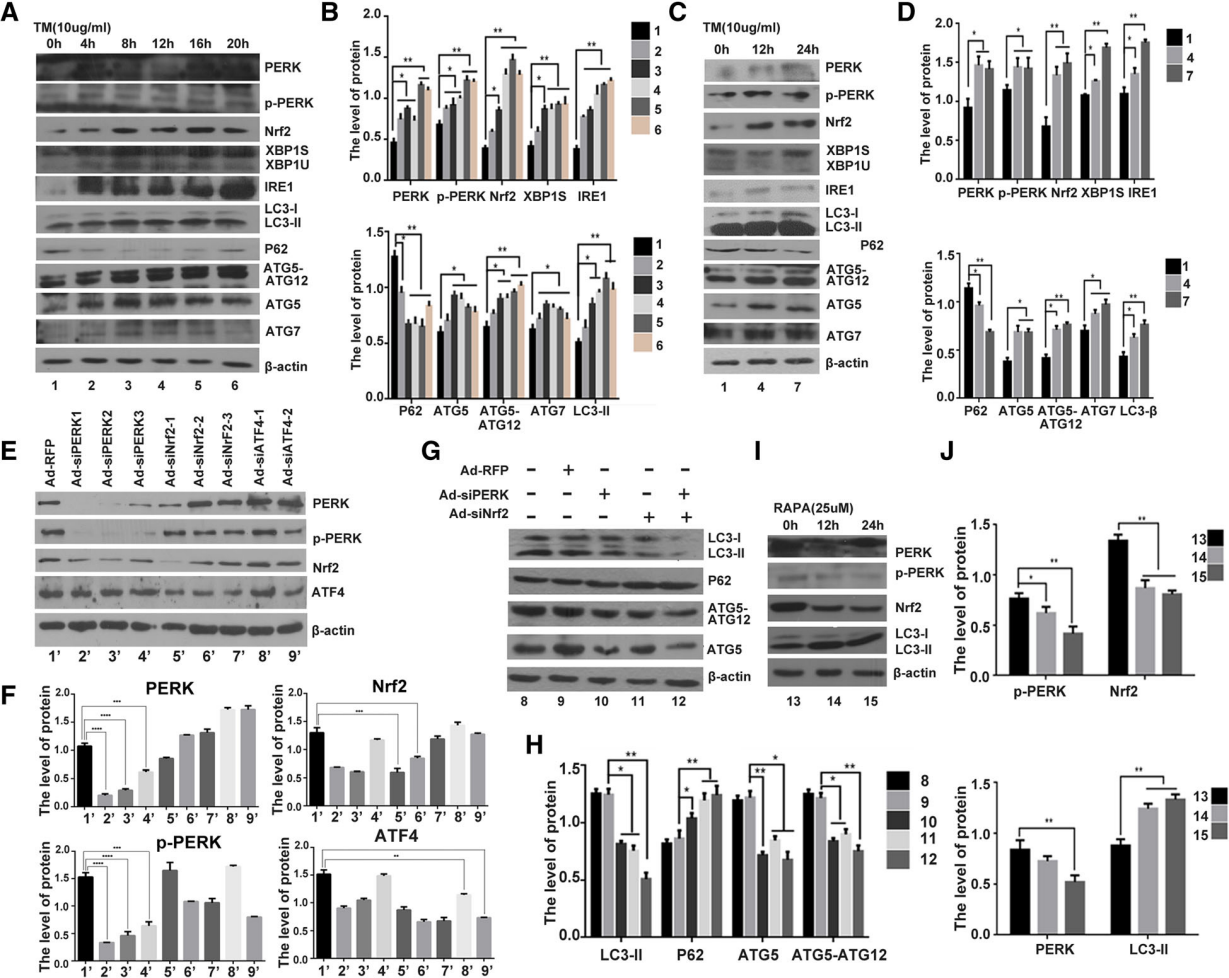
Expression of ER stress associated protein and autophagy related protein induced by Tm or RAPA in human chondrocyte. **a** C28I2 cells were incubated with 10 μg/ml tunicamycin (Tm), a typical ER stress inducer, with the time intervals (0, 4, 8, 12, 16, 20 h). ER stress related proteins and autophagy associated proteins were analysed by western blotting. **b** Qualitative analysis of related proteins. The values were normalized to β-actin (Bio-Rad), as indicated, data were expressed as means±S.D. *n* = 3). Every treatment group compared with control groups, respectively, *P < 0.05, **P < 0.01. **c** C28I2 cells were treated with TM after 12 h and 24 h. ER stress related proteins and autophagy proteins were analysed by western blotting. Similar method with before. **d** The levels of related proteins were normalized to β-actin, data were expressed as means ± S.D. *n* = 3). Every treatment group compared with control groups, respectively, *P < 0.05, **P < 0.01. **e** Determination the expression of PERK,Nrf2 and ATF4 by western blotting after infected with siPERK1/2/3, siNrf2–1/2/3 and siATF4–1/2 in C28I2 cells. **f** The levels of related proteins were normalized to β-actin, data were expressed as means±S.D. *n* = 3). Every treatment group compared with control groups, respectively, *P < 0.05, **P < 0.01. **g** Determination of autophagy proteins expression by western blotting after infected with siPERK1, siNrf2–1in C28I2 cells. **h** The levels of related proteins were normalized to β-actin, data were expressed as means±S.D. *n* = 3). Every treatment group compared with control groups, respectively, *P < 0.05, **P < 0.01. **i** C28I2 cells were incubated with rapamycin(RAPA) (25 μM), a typical autophagy inducer, after 12 h and 24 h. ER stress related protein PERK, p-PERK and Nrf2 were detected by western blotting. **j** The levels of related proteins were normalized to β-actin. Every treatment group compared with control groups, respectively. Values are means ± SD (n = 3), *P < 0.05, **P < 0.01. (1:TM 0 h; 2:TM 4 h;3:TM 8 h;4:TM 12 h;5:TM 16 h; 6:TM 20 h; 7:TM 24 h; 8:NC;9: Ad-RFP(siRNA control); 10:Ad-siPERK; 11:Ad-siNrf2; 12: siPERK+siNrf2;13:RAPA 0 h;14:RAPA 12 h;15:RAPA 24 h. 1′:Ad-RFP, 2′:Ad-siPERK1,3′:Ad-siPERK2,4′:Ad-siPERK3, 5′:Ad-siNrf2–1, 6′:Ad-siNrf2–2; 7′:Ad-siNrf2–3; 8′:Ad-siATF4–1; 9′: Ad-siATF4–2)

It is well known that when UPR is triggered in ER stress, the activation of PERK signaling pathway is initiated upon its dimerization and autophosphorylation [20–22]. Tunicamycin (Tm), as a typical ER stress inducer, can obviously elicit the ER stress, such as accumulation of PERK(125KD), phosphorylated PERK (125KD), nuclear transcription factor Nrf2 (68KD), IRE1 (110KD) and XBP1s/XBP1u (40KD/29KD) [35, 36]. We detected the expression of autophagy related proteins under ER stress induced by Tm. The result showed that the ER stress-associated molecules, were activated in Tm-treated cells (Fig. 1a, c), and the expression of autophagy related proteins, including ATG5 (33KD), ATG5-ATG12 (56KD), ATG7 (78KD), LC3-I/LC3-II (16KD/18KD) were also increased, the expression of P62 (62KD) was reduced after Tm treatment (Fig. 1a, b, c and d). Furthermore, we detected the expression of autophagy related proteins during ER stress inhibited by siPERK and siNrf2, the specific siRNA approach. The inhibit rate of siPERK1, siPERK2 and siPERK3 are 78, 69 and 45% respectively. The inhibit rate of siNrf2–1, siNrf2–2 and siNrf2–3 are 65, 58 and 21% respectively. The inhibit rate of siATF4–1, siATF4–2 are 38 and 56% respectively (Fig. 1e, f).

The result showed that the expression of ATG5, ATG7, LC3-I/LC3-II were obviously reduced, and the expression of P62 was increased after siPERK and siNrf2 treatment. Knockdown expression of PERK and Nrf2 can inhibit autophagy activation(Fig. 1g, h). We then detected ER stress associated proteins expression after rapamycin (RAPA), a typical autophagy inducer, treatment, and it is showed that PERK, p-PERK and Nrf2 were depressed in the RAPA-treated chondrocyte (Fig. 1i and j).These results suggested that autophagy affected ER stress in human chondrocyte, and vice versa.

### Overexpression of ATG5 or ATG7 enhance autophagy and inhibit ER stress in chondrocyte

**Fig. 2:**
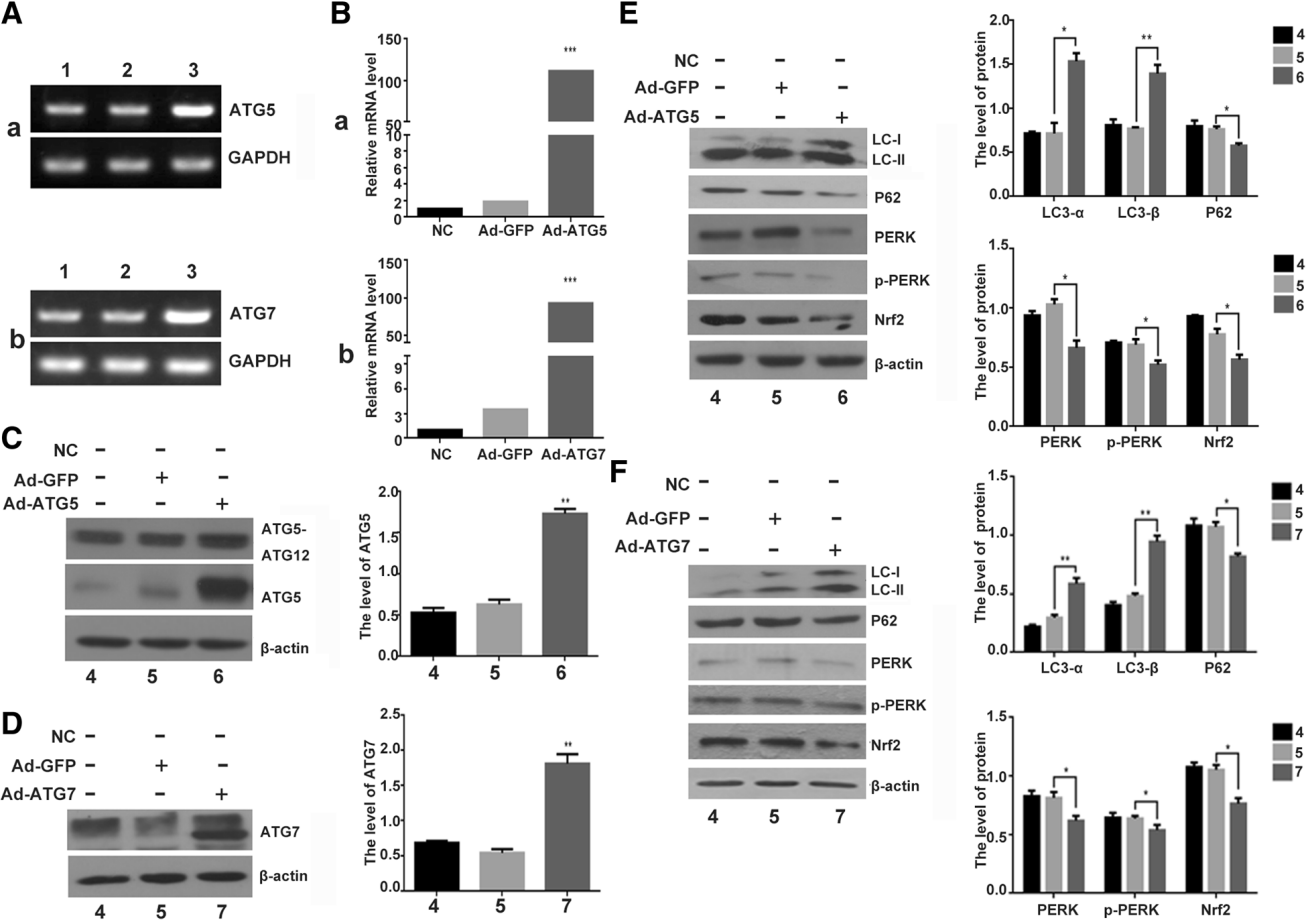
Expression of ER stress associated protein and autophagy related protein in the C28I2 chondrocyte infected with Ad-ATG5 or Ad-ATG7. **a** The ATG5 and ATG7 mRNA level were analysed by RT-PCR in C28I2 cells. **b** Analysis of ATG5 and ATG7 mRNA level with Q-PCR in C28I2 cells. Ad-ATG5(a), Ad-ATG7(b). **c** The ATG5 protein expression was analysed by western blotting in the Ad-ATG5 infected C28I2 cells. The level of ATG5 was normalized to β-actin. **d** The ATG7 protein expression was analysed by western blotting in the Ad-ATG7 infected C28I2 cells. The level of ATG7 was normalized to β-actin. **e** The expression levels of LC3, P62, PERK, p-PERK, Nrf2 proteins after infected with Ad-ATG5 in C28I2 cells for 24 h by western blotting. The levels of related proteins were normalized to β-actin. **f** The expression levels of LC3, P62, PERK, p-PERK, Nrf2 proteins after infected with Ad-ATG7 in C28I2 cells for 24 h by western blotting. The levels of related proteins were normalized to β-actin. *P < 0.05, **P < 0.01, ***P < 0.001 compared with the controls. Values are means±SD *n* = 3). (1:NC, 2: Ad-GFP, 3:Ad-ATG5(a) and Ad-ATG7(b), 4:NC, 5:Ad-GFP, 6:Ad-ATG5, 7:Ad-ATG7)

To detect the effect of ER stress by ATG5 and ATG7, two critical autophagy related proteins, Ad-ATG5 and Ad-ATG7 adenoviruses vectors were constructed and identified with endonuclease digesting and DNA sequencing, respectively. The results showed that the construction of plasmids were correct (Fig. 1s, Additional file 1). Then the C28I2 chondrocytes infected with Ad-ATG5 or Ad-ATG7 were identified by RT-PCR,Q-PCR and Western blot. The level of ATG5 and ATG7 mRNA were obviously increased in the Ad-ATG5 and Ad-ATG7 infected cells, comparing with their controls, Ad-GFP as a control (Fig. 2a, b). And the protein levels were also significantly enhanced in the Ad-ATG5 and Ad-ATG7 infected cells, comparing with the other two control cells, respectively (Fig. 2c, d). The results illustrated that the construction and expression of Ad-ATG5 and Ad-ATG7 were correct.

We next to examine the expression of autophagy related protein, including LC3, P62, and ER stress associated protein, such as PERK, p-PERK, Nrf2, in the Ad-ATG5 and Ad-ATG7 infected chondrocytes. The result showed that LC3-I/LC3-II were increased and P62 were reduced in the Ad-ATG5 and Ad-ATG7 infected chondrocytes comparing with the controls. Furthermore, the expression of PERK、p-PERK and Nrf2 were decreased in the Ad-ATG5 and Ad-ATG7 infected chondrocytes comparing with the controls (Fig. 2e and f). Taken together, overexpression of ATG5 or ATG7 enhance autophagy and inhibit ER stress in chondrocyte.

### The effect of ATG5 and ATG7 on autophagy and ER stress in chondrocyte

**Fig. 3:**
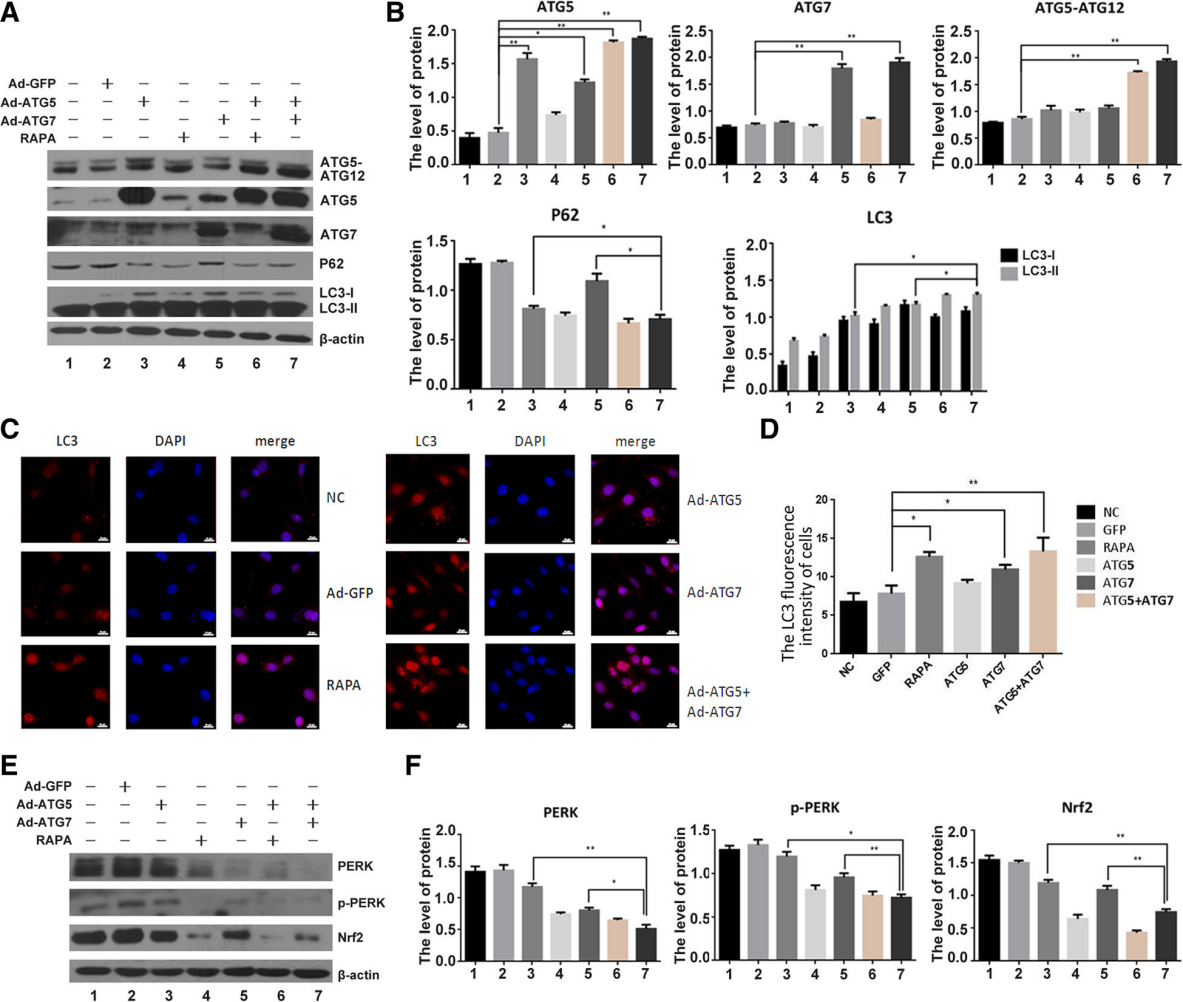
ATG5 and ATG7 enhanced autophagy and inhibited ER stress in chondrocyte. **a** Western blotting analysis of LC3, P62, ATG5, ATG7 and ATG5-ATG12 expression after infected with Ad-ATG5, Ad-ATG7 and Ad-ATG5 + Ad-ATG7 in the C28I2 cells. β-actin is served as an internal control. **b** Qualitative analysis of ATG5, ATG7, ATG5-ATG12, LC3 and P62. The values were normalized to β-actin. **c** C28I2 cells were double stained with LC3 (red) and DAPI (blue) and visualized by confocal microscopy (400X) after treated with Rapamycine, Ad-ATG5, Ad-ATG7 and Ad-ATG5 + Ad-ATG7 24 h. *P < 0.05, **P < 0.01 compared with the controls. Values are means ± SD *n* = 3). **d** Qualitative analysis of LC3 fluorescence intensity of chondrocytes. The values were normalized to the NC group. **e** Western blotting analysis of PERK, p-PERK and Nrf2 expression after infected with Ad-ATG5, Ad-ATG7 and Ad-ATG5 + Ad-ATG7 in the C28I2 cells. β-actin is served as an internal control. **f** Qualitative analysis of PERK, p-PERK and Nrf2 were normalized to β-actin. (1:NC, 2:Ad-GFP, 3:Ad-ATG5, 4:RAPA, 5:Ad-ATG7, 6:Ad-ATG5 + RAPA, 7:Ad-ATG5 + Ad-ATG7). Rapamycin (25 μM) used as a positive control

Next, we examined how the ATG5 and ATG7 influence on autophagy and ER stress. As revealed in Fig. 3a and b, The level of autophagy proteins, ATG5, ATG7, ATG5-ATG12 and LC3-I/LC3-II were significantly increased after infected with Ad-ATG5, Ad-ATG7, Ad-ATG5 + Ad-ATG7 in chondrocyte, and ATG5 + ATG7 improved this enhancement furtherly. In addition, we detected the combined effect of ATG5 and ATG7 on the expression of PERK, p-PERK and Nrf2, three kinds of ER stress related proteins. The expression of PERK, p-PERK and Nrf2 were obviously reduced after infected with Ad-ATG5, Ad-ATG7, Ad-ATG5 + Ad-ATG7 comparing with the control, and the combined ATG5 and ATG7 aggravated this repression effect. Rapamycin used as a positive control (Fig. 3e and f).

The immunofluorescence images showed that not only Ad-ATG5 or Ad-ATG7 enhanced the LC3 expression respectively, but also their combined effect significantly increased the LC3 expression comparing with their individual effect (Fig. 3c, d). Taken together, the individual effect of ATG5 and ATG7 increased autophagy and reduced ER stress, the combined of them could obviously improve the effect of autophagy enhancement and ER stress inhibition. The augment of autophagy-related protein ATG5 and ATG7 may partially abolish ER stress activation.

### The effect of ATG5 and ATG7 on autophagy flux and autolysosome formation

It is known that lysosome-associated membrane proteins 1 and 2 (LAMP-1 and LAMP-2), as the major protein components of the lysosomal membrane, are delivered to phagosomes during autophagy process. Microtubule-associated protein 1α/β-light chain 3 (LC3) is a kind of soluble protein, which is recruited to autophagosomal membranes during autophagy process. Autophagosomes fuse with lysosomes to form autolysosomes, and intra-autophagosomal components are degraded by lysosomal hydrolases [37–39].

**Fig. 4:**
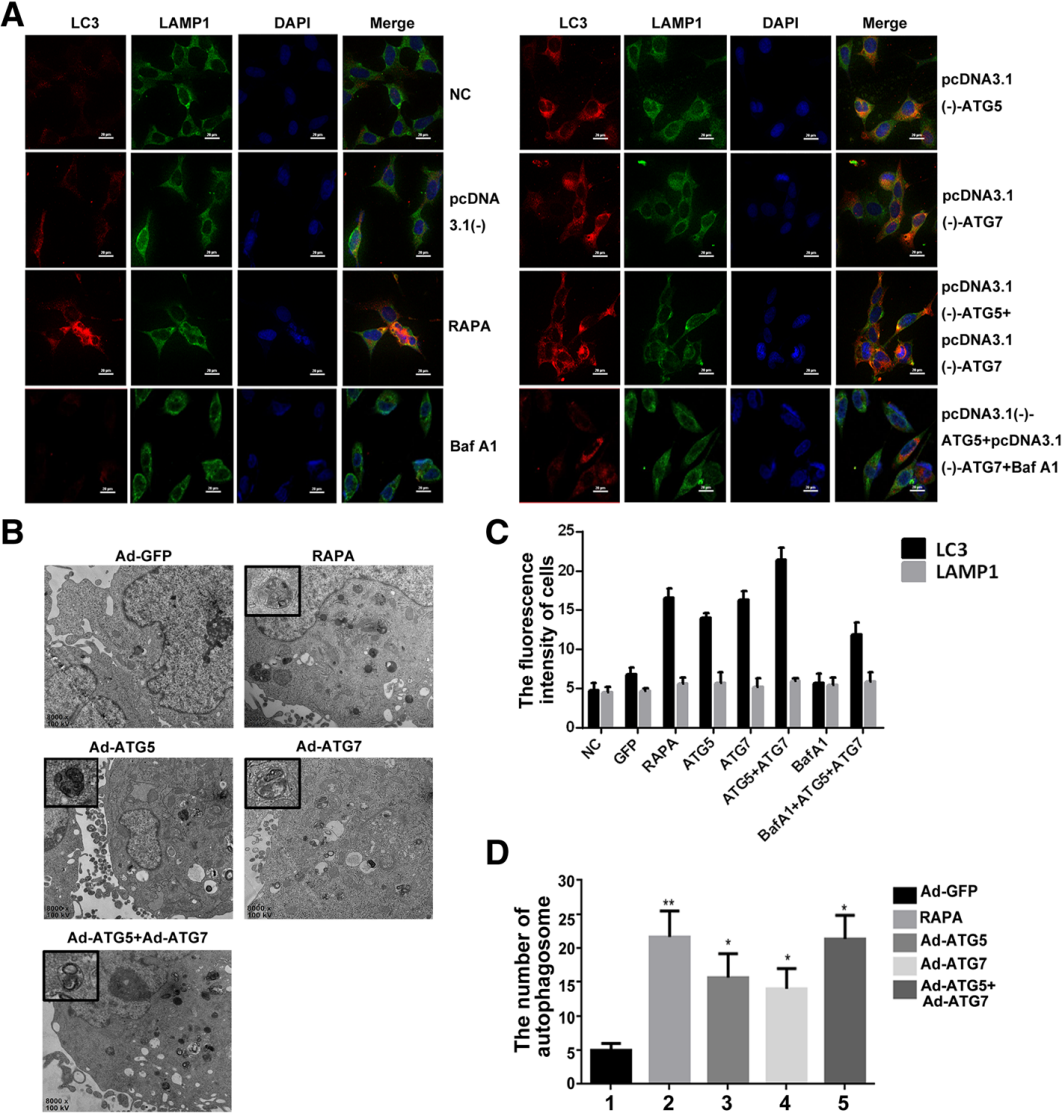
Determination the effect of ATG5 and ATG7 on autophagy flux. **a** The C28I2 cells were treated with Rapamycin (25 μM), pcDNA3.1(−), pcDNA3.1(−)-ATG5, pcDNA3.1(−)-ATG7, pcDNA3.1(−)-ATG5 + pcDNA3.1(−)-ATG7, Bafilomycin A1(0.4 μM), and Bafilomycin A1(0.4 μM) + pcDNA3.1 (−)-ATG5 + pcDNA3.1(−)-ATG7, then immediately stained with the anti-LC3 antibody, anti-LAMP1 antibody and DAPI respectively, and visualized by confocal microscopy (400×). **b** Transmission electron microscopy (TEM) analysis showing autophagosome (arrowed) after treated with Rapamycin (25 μM), Ad-GFP, Ad-ATG5, Ad-ATG7 and Ad-ATG5 + Ad-ATG7 for 24 h in the C28I2 cells. The autophagosome is shown by red arrow. Rapamycin (25 μM) used as a positive control, Bafilomycin A1 is a lysosomal inhibitor. **c** Qualitative analysis of LC3 and LAMP1 fluorescence intensity of chondrocytes under confocal microscopy. The values were normalized to the NC group. **d** Qualitative analysis of the number of autophagosome in chondrocytes under TEM. The values were normalized to the NC group

In order to assay for autophagic flux, we detected whether autophagosome fused with lysosome and formed autolysosomes with red fluorescent-tagged LC3 and green fluorescent-tagged LAMP1. Briefly, the C28I2 cells were treated with Rapamycin (25 μM), pcDNA3.1(−), pcDNA3.1(−)-ATG5, pcDNA3.1(−)-ATG7 and pcDNA3.1(−)- ATG5 + pcDNA3.1(−)-ATG7, Bafilomycin A1(0.4 μM), Bafilomycin A1 + pcDNA3.1 (−)-ATG5 + pcDNA3.1(−)-ATG7, respectively. Then incubated with anti-LC3 antibody, anti-LAMP1 antibody and DAPI respectively. Rapamycin, a basic of autophagy inducer, can improve autophagic flux formation. Bafilomycin A1, a basic lysosomal inhibitor, can prevent the fusion of autophagosome and lysosome. The result showed that the individual treatment of ATG5 or ATG7 can increase the autolysosome formation comparing with the control. Meanwhile, the combined effect of pcDNA3.1(−)-ATG5 + pcDNA3.1(−)-ATG7 can significantly improve this enhancement effect and increase the autophagy flux furtherly, however, the augment effect of pcDNA3.1(−)-ATG5 + pcDNA3.1(−)-ATG7 can be blocked after Bafilomycin A1 treatment (Fig. 4a, c).

Next, from the transmission electron microscope (TEM) images, the autophagosome and the autolysosome were dramaticlly increased in the Ad-ATG5 + Ad-ATG7 group compared with that of the Ad-GFP control and the individual treatment of ATG5 or ATG7. Rapamycin used as a positive control (Fig. 4b, d). These results indicated that the individual ATG5 or ATG7 could increase autophagy flux and ATG5 + ATG7 can clearly enhance this effect and augment the autolysosome formation furtherly in chondrocytes.

### The effect of ATG5 and ATG7 on cell apoptosis and cell proliferation in chondrocyte

**Fig. 5:**
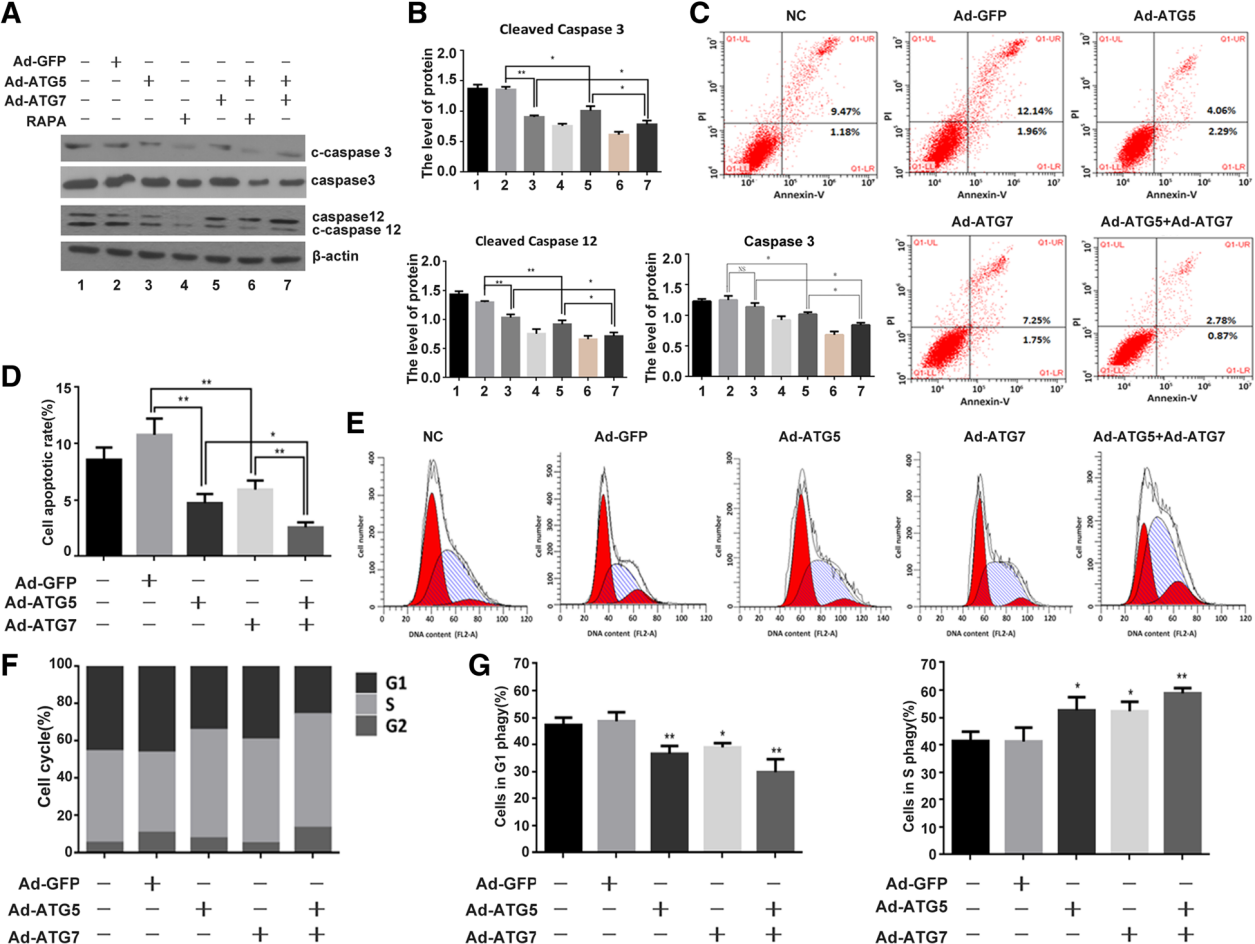
ATG5 and ATG7 inhibited apoptosis and promoted chondrocyte proliferation. **a** The expression of caspase3, cleaved caspase3 and cleaved caspase12 protein were analysed by western blotting after infected with Ad-GFP, Ad-ATG5, Ad-ATG7 and Ad-ATG5 + Ad-ATG7 in C28I2 cells. **b** Qualitative analysis of caspase3, cleaved caspase3 and cleaved caspase12. The values were normalized to β-actin. Rapamycin (25 μM) used as a positive control. **c** Flow cytometry (FCM) analysis with Annexin V –PI staining was performed to evaluate the percentage of apoptotic cells in C28I2 cells after infected with Ad-GFP, Ad-ATG5, Ad-ATG7 and Ad-ATG5 + Ad-ATG7. The percentage of apoptotic cells in the Ad-ATG5 + Ad-ATG7 group was significantly reduced compared with that of the Ad-GFP, the Ad-ATG5 and the Ad-ATG7 group. **d** Analysis on cell apoptosis results. Data are mean ± SD for relative apoptosis normalized to control cells for three independent experiments. Columns mean of five separate experiments. Representative images from flow cytometry analysis are shown. **e** Flow cytometry images with propidium iodide (PI) staining and analysis of cell cycle distribution with flow cytometry. Experiments were repeated three times, samples were analyzed by Student’s t test and statistical significance with *P* < 0.05. Representative images were shown. Experiments were repeated three times. **f** Percentage of cells at each phase in C28I2 cells after infected with Ad-GFP, Ad-ATG5, Ad-ATG7 and Ad-ATG5 + Ad-ATG7. **g** Percentage of S phase and G1 phase in C28I2 cells infected with Ad-ATG5, Ad-ATG7, Ad-ATG5 + Ad-ATG7.*P < 0.05, **P < 0.01 compared with the controls. Values are means ± SD *n* = 3). (1:NC, 2:Ad-GFP, 3:Ad-ATG5, 4:RAPA, 5:Ad-ATG7, 6:Ad-ATG5+ RAPA, 7:Ad-ATG5 + Ad-ATG7)

Next, we sought to determine the effects of Ad-ATG5, Ad-ATG7 and Ad-ATG5 + Ad-ATG7 on ER stress-mediated cell apoptosis and cell cycle. As revealed in Fig. 5a and b, the expressions of cleaved caspase3 (16KD/18KD) and cleaved caspase12 (42KD) were markedly reduced in the C28I2 cells infected with Ad-GFP, Ad-ATG5, Ad-ATG7 and Ad-ATG5 + Ad-ATG7. Rapamycin used as a positive control. It is suggested that not only Ad-ATG5 and Ad-ATG7 reduce apoptosis respectively, but also their combined effect can dramaticlly inhibit apoptosis compared with that of the control, the Ad-ATG5 and the Ad-ATG7 individually. Furthermore, FCM result also showed that the apoptosis rate markedly decreased in the C28I2 cells infected with Ad-ATG5 (6.35%), Ad-ATG7 (9.00%) and Ad-ATG5 + Ad-ATG7 (3.65%) compared with that of the NC control (10.65%) and the Ad-GFP control (14.1%, Fig. 5c and d). The differences between each treatment group are of statistical significance (*P* < 0.05). The cell cycle distribution was analyzed by Flow cytometry analysis (FCM) as shown in Fig. 5e, f and g. The data showed that the S phase proportion of C28I2 cells infected with Ad-ATG5 (58.12%), Ad-ATG7 (55.63%) and Ad-ATG5 + Ad-ATG7 (61.03%) clearly increased compared with that of the Ad-GFP control (42.98%). The G1 phase cell numbers were Ad-GFP (46.29%), Ad-ATG5 (34.22%), Ad-ATG7 (39.28%) and Ad-ATG5 + Ad-ATG7 (25.71%) respectively. It is suggested that the individual ATG5 or ATG7 can promote cells from G1 phase to S phase and inhibit ER stress-mediated apoptosis in chondrocyte. Futhermore,their combined effect can significantly increase the cell number in S phase and thus dramatically promote this cell proliferation enhancement and ER stress-mediated apoptosis repression.. The differences between treatment groups and control groups have statistical significance (*P* < 0.05).

### The effect of ATG5 and ATG7 on autophagy, ER stress, apoptosis and cell cycle through PERK signaling

**Fig. 6:**
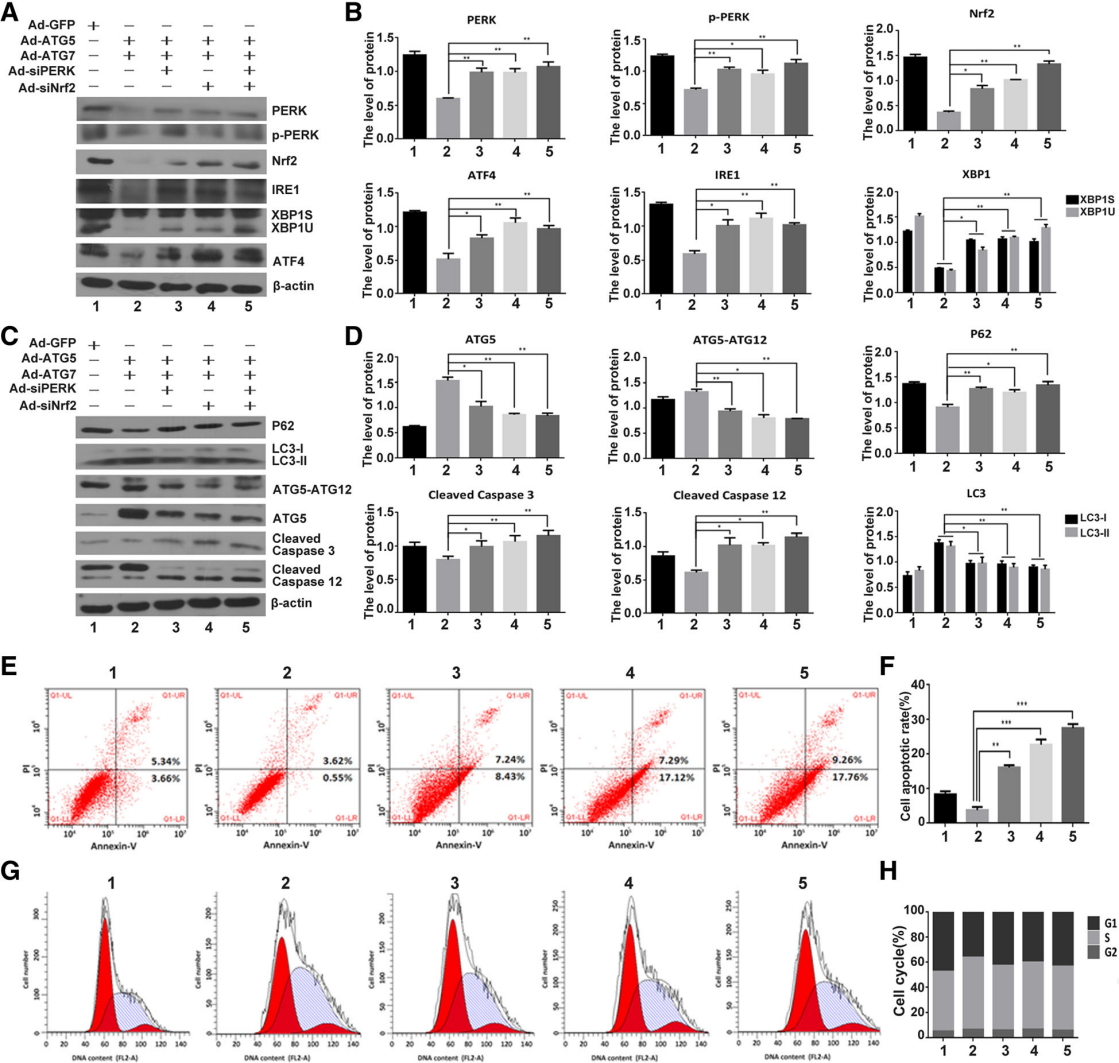
ATG5 and ATG7 influenced on autophagy, apoptosis and cell cycle through PERK/Nrf2 signaling. **a** The ER stress proteins were analysed by western blotting in Ad-GFP, Ad-ATG5 + Ad-ATG7,Ad-ATG5 + Ad-ATG7 + siPERK,Ad-ATG5 + Ad-ATG7 + siNrf2 and Ad-ATG5+ Ad-ATG7 + siPERK+siNrf2 induced chondrocytes. **b** The levels of ER stress proteins were normalized to β-actin. **c** Determination of autophagy and apoptosis proteins expression by western blotting in Ad-GFP, Ad-ATG5 + Ad-ATG7, Ad-ATG5 + Ad-ATG7 + siPERK,Ad-ATG5 + Ad-ATG7 + siNrf2 and Ad-ATG5+ Ad-ATG7 + siPERK+ siNrf2 induced chondrocytes. **d**The levels of related proteins were normalized to β-actin. **e** FCM analysis was used to calculate the percentage of apoptotic cells at the time point of 24 h. The apoptosis rate were increased when combined treatment with silencing of PERK or Nrf2. Experiments were repeated 3 times, Representative images are shown. **f **Analysis of cell apoptosis. Data come from 3 independent experiments. **g** FCM analysis indicated that the S phase percentage were decreased compared to that of the control groups when combine infected with silencing of PERK or Nrf2 in C28I2 cells. Experiments were repeated 3 times, Representative images are shown. **h** Percentage of cells at each phase in different groups. *P < 0.05, **P < 0.01 compared with the controls. Values are means ± SD *n* = 3). (1:Ad-GFP, 2:Ad-ATG5 + Ad-ATG7, 3:Ad-ATG5 + Ad-ATG7 + siPERK, 4:Ad-ATG5 + Ad-ATG7 + siNrf2, 5:Ad-ATG5 + Ad-ATG7 + siPERK+ siNrf2)

Autophagy as a cytoprotective mechanism to reduce the damaged organelles or degraded macromolecules in cells [40, 42]. To further explore which UPR signal pathway is involved in autophagy and ER stress-mediated apoptosis, the expressions of PERK, p-PERK, Nrf2, IRE1, XBP1s/u, ATG5, LC3-I/LC3-II, P62 and ATG5-ATG12 were examined in the C28I2 cells infected with Ad-GFP, Ad-ATG5 + Ad-ATG7, Ad-ATG5 + Ad-ATG7 + Ad-siPERK, Ad-ATG5 + Ad-ATG7 + Ad-siNrf2, Ad-ATG5 + Ad-ATG7 + Ad-siPERK+Ad-siNrf2. As revealed in Fig. 6a and b, ATG5 + ATG7 increased autophagy and inhibited ER stress, however, the effect of ATG5 + ATG7 on ER stress repression was vanished in the C28I2 cells treated with siPERK, siNrf2, siPERK+siNrf2,as revealed by enhanced expressions of PERK, p-PERK,Nrf2, IRE1, XBP1s/u.On the other side,the effect of ATG5 + ATG7 on autophagy enhancement was decreased in the C28I2 cells treated with siPERK, siNrf2, siPERK+siNrf2, as revealed by expressions of ATG5, LC3-I/LC3-II, P62 and ATG5-ATG12. Furthermore, after treated with siPERK, siNrf2,siPERK+siNrf2, the effect of ATG5 + ATG7 on apoptosis inhibition was eliminated, as shown by expressions of cleaved caspase3 and cleaved caspase12 (Fig. 6c and d). FCM result also confirmed this result. The cell apoptotic rate was 4.17% in the C28I2 cells infected with Ad-ATG5 + Ad-ATG7, and increased in the C28I2 cells treated with Ad-ATG5 + Ad-ATG7 + siPERK (15.67%), Ad-ATG5 + Ad-ATG7 + siNrf2 (24.41%), Ad- ATG5 + Ad-ATG7 + siPERK+siNrf2 (27.02%) (Fig. 6e and f). It is demonstrated that ATG5 and ATG7 increased autophagy, inhibit ER stress and apoptosis through PERK/Nrf2 signaling. Moreover, we detected cell cycle distribution of C28I2 cells in the presence of Ad-ATG5 + Ad-ATG7 + siPERK, Ad-ATG5 + Ad-ATG7 + siNrf2 and Ad-ATG5 + Ad-ATG7 + siPERK+siNrf2. The S phase cell number was 57.31% in the C28I2 cells treated with Ad-ATG5 + Ad-ATG7, and reduced in the C28I2 cells treated with Ad-ATG5 + Ad-ATG7 + siPERK (51.22%), Ad-ATG5 + Ad-ATG7 + siNrf2 (53.19%), Ad-ATG5 + Ad-ATG7 + siPERK+siNrf2 (50.87%), respectively. The data demonstrated that the effect of ATG5 + ATG7 on cell growth enhancement was decreased in the C28I2 cells treated with siPERK, siNrf2 and siPERK+siNrf2. The differences between the S phase cell number were up to statistical significance (*P* < 0.05) (Fig. 6g and h).

**Fig. 7:**
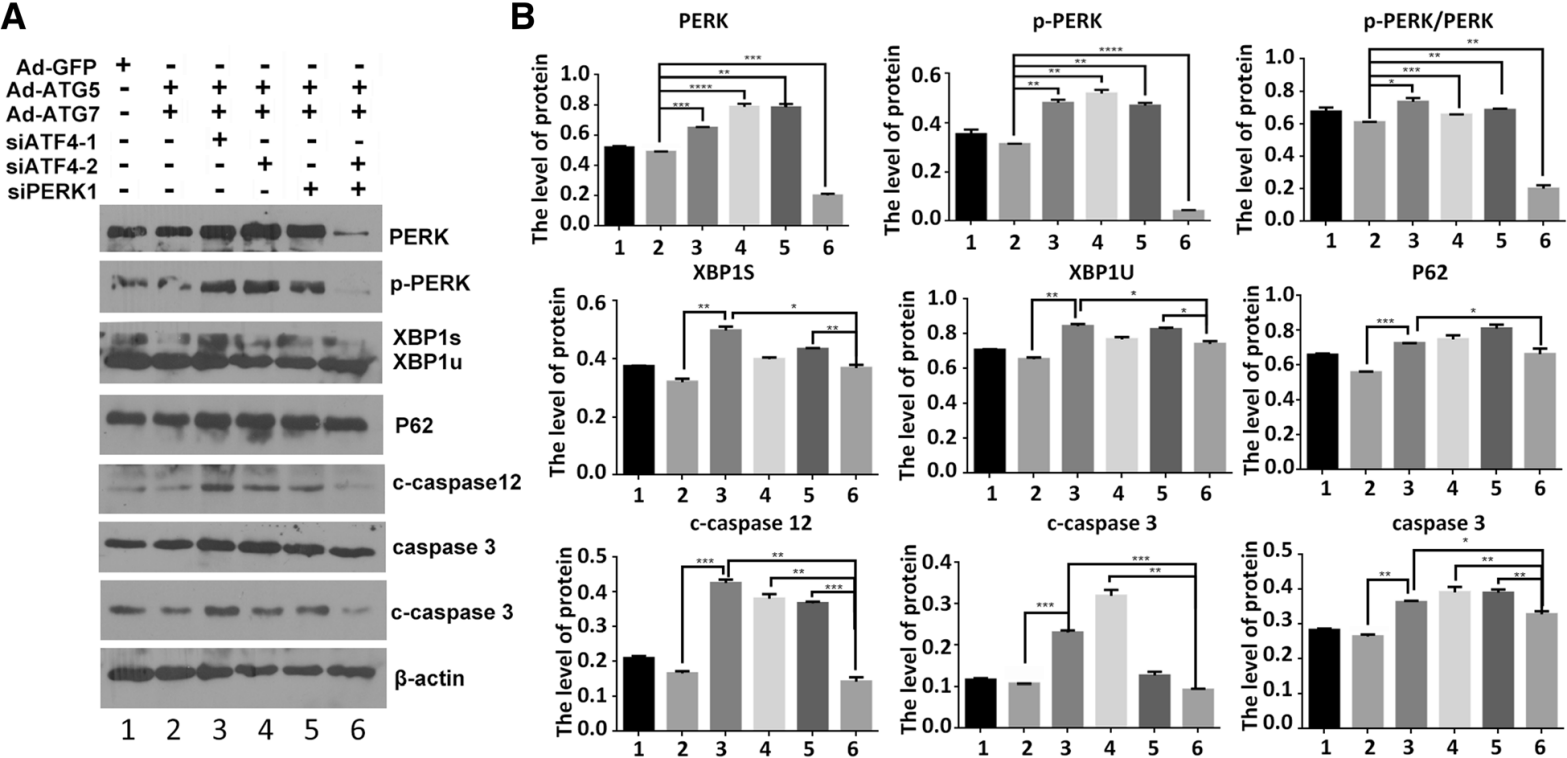
ATG5 and ATG7 influenced on autophagy, apoptosis through PERK/ATF4 signaling. **a** The ER stress proteins were analysed by western blotting in Ad-GFP, Ad-ATG5 + Ad-ATG7,Ad-ATG5 + Ad-ATG7 + siATF4–1,Ad-ATG5 + Ad-ATG7 + siATF4–2,Ad-ATG5 + Ad-ATG7 + siPERK1 and Ad-ATG5 + Ad-ATG7 + siPERK1+siATF4–2 induced chondrocytes. **b** The levels of related proteins were normalized to β-actin. (1:Ad-GFP, 2:Ad-ATG5 + Ad-ATG7, 3:Ad-ATG5 + Ad-ATG7 + siATF4–1, 4:Ad-ATG5 + Ad-ATG7+ siATF4–2, 5:Ad-ATG5 + Ad-ATG7 + siPERK1, 6:Ad-ATG5 + Ad-ATG7 + siPERK1+ siATF4–2)

All is known that ATF4 is another downstream molecule of PERK, we also detected whether the effect of ATG5 and ATG7 is associated with ATF4. As revealed in Fig. 7a and b, the effect of ATG5 + ATG7 on ER stress repression was vanished in the siATF4-treated chondrocytes, however, this inhibition effect of siATF4 is blocked after siPERK treatment. On the other side, siATF4 decrease the effect of ATG5 + ATG7 on autophagy enhancement and apoptosis inhibition, and this effect of siATF4 is eliminated by siPERK (Fig. 7a and b).

Taken together, these results manifest the effect of ATG5 and ATG7 on autophagy, ER stress, cell cycle and apoptosis through PERK signalling. The protective effect of ATG5/7 overexpression on chondrocyte survival is dependent upon PERK signaling. Interestingly, the effect of siPERK and siNrf2 on the cytoprotective effect of ATG5/7 are of synergism, while the effect of siPERK and siATF4 are of antagonism. PERK signal may be the pivot for autophagy and ER stress.

### PERK is required for the effect of ATG5 and ATG7 on autophagy, ER stress, apoptosis and cell cycle

**Fig. 8:**
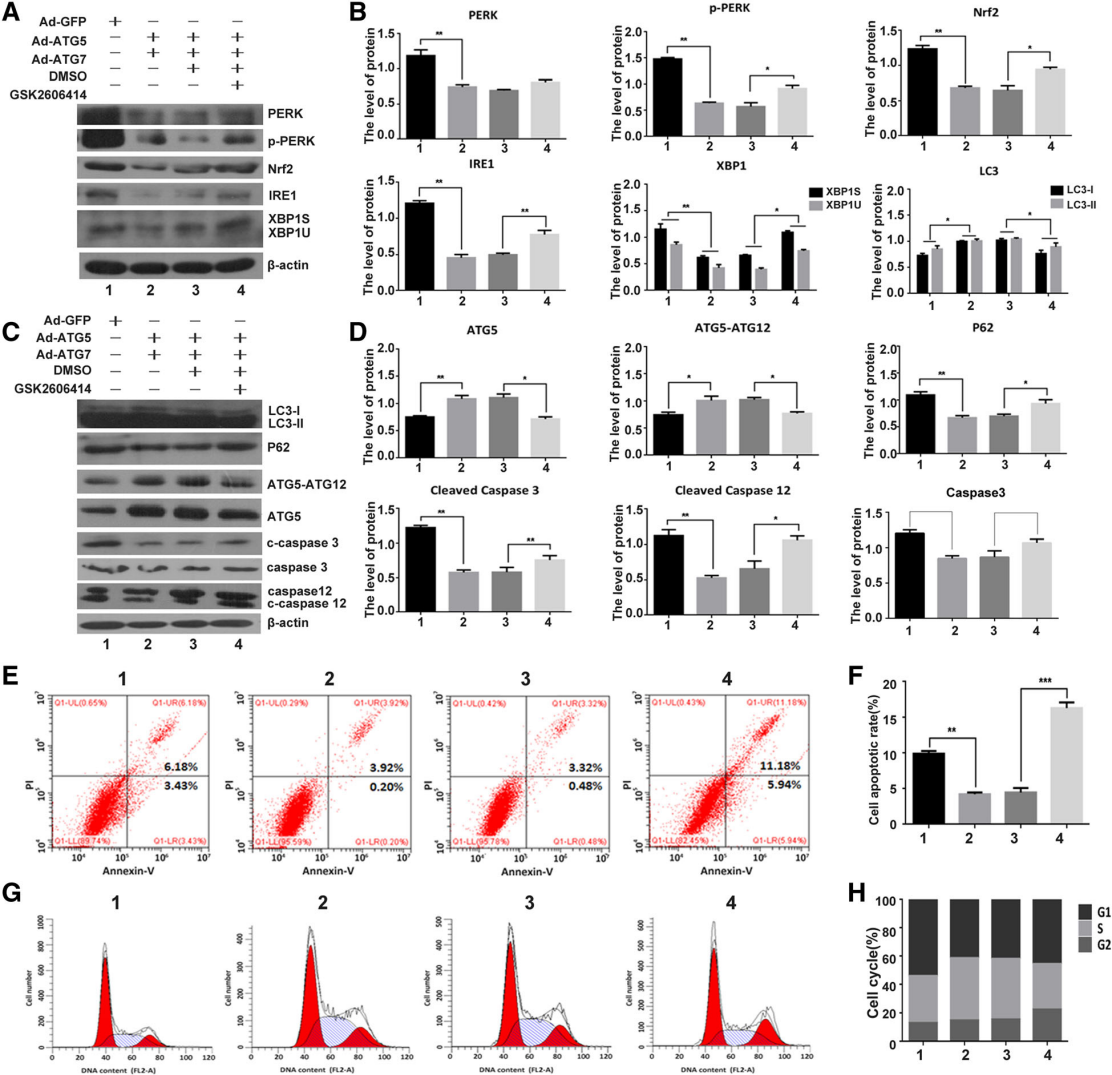
The effect of ATG5 and ATG7 on autophagy, ER stress, apoptosis and cell cycle depends on PERK. **a** The ER stress proteins were analysed by western blotting in the GSK2606414 treated chondrocytes. **b** The levels of ER stress proteins were normalized to β-actin. **c** Determination of autophagy and apoptosis proteins expression by western blotting in the GSK2606414 treated chondrocytes. **d** The levels of related proteins were normalized to β-actin. **e** FCM analysis was used to calculate the percentage of apoptotic cells at the time point of 24 h. The apoptosis rate were increased when combined treated with GSK2606414. Experiments were repeated 3 times, Representative images are shown. **f** Analysis of cell apoptosis. Data come from 3 independent experiments. **g** FCM analysis indicated that the S phase percentage were decreased compared to that of the control groups when combine treated with GSK2606414 in C28I2 cells. Experiments were repeated 3 times, Representative images are shown. **h** Percentage of cells at each phase in different groups. *P < 0.05, **P < 0.01 compared with the controls. Values are means ± SD *n* = 3). (1:Ad-GFP, 2:Ad-ATG5 + Ad-ATG7, 3:Ad-ATG5 + Ad-ATG7 + DMSO, 4:Ad-ATG5 + Ad-ATG7+ GSK2606414)

We next used GSK2606414, a typical PERK pathway inhibitor, to validate the effect of ATG5 and ATG7 on autophagy and ER stress. As shown in Fig. 8a, b, c and d, ATG5 + ATG7 increased autophagy and inhibited ER stress, however, the effect of ATG5 + ATG7 on ER stress repression was vanished after treated with GSK2606414, as revealed by enhanced expressions of PERK, p-PERK, Nrf2, IRE1, XBP1s/u. On the other side, the effect of ATG5 + ATG7 on autophagy enhancement was reduced after treated with GSK2606414, as revealed by expressions of ATG5, LC3-I/LC3-II, P62 and ATG5-ATG12. Furthermore, after treated with GSK2606414, the effect of ATG5 + ATG7 on apoptosis inhibition was eliminated, as shown by enhanced expressions of cleaved caspase3 and cleaved caspase12 (Fig. 8c and d). FCM result also proved this result. The cell apoptotic rate was 4.12% in the Ad-ATG5 + Ad-ATG7 infected C28I2 cells, and increased in the C28I2 cells treated with Ad-ATG5 + Ad-ATG7 + GSK2606414 (17.12%) (Fig. 8e and f). The data demonstrated that ATG5 and ATG7 regulate autophagy, ER stress and apoptosis through PERK signaling.

Moreover, we detected cell cycle distribution of C28I2 cells in the presence of Ad-ATG5 + Ad-ATG7 and Ad-ATG5 + Ad-ATG7 + GSK2606414. The S phase cell number was 43.87% in the C28I2 cells treated with Ad-ATG5 + Ad-ATG7, and reduced in the C28I2 cells treated with Ad-ATG5 + Ad-ATG7 + GSK2606414 (32.00%). The proportion of S phase were decreased after GSK2606414 treatment (Fig. 8g and h). It is showed that ATG5 and ATG7 promote cell proliferation through PERK signaling. The differences between the S phase cell number were up to statistical significance (*P* < 0.05). Collectively, The effects of ATG5 and ATG7 on autophagy, ER stress, apoptosis and cell cycle were through PERK signalling pathway.

## Discussion

Cell survival, proliferation, autophagy and apoptosis are intimately connected processes, which are regulated by the mammalian target of rapamycin (mTOR) kinase and the ER stress pathway, also known as the UPR [43, 44]. As many literature reported, there are many connections between UPR and autophagy, such as, ATF6a branch which, as one of UPR pathway, is involved in the activation of mTORC1 and PERK which, as a major transducer of the ER stress, can mediate transcriptional activation of LC3 and ATG5 proteins in hypoxia. ATG5 and LC3 are involved in phagophore expansion and autophagosome formation [45–48]. Selective autophagy of the ER—ER-phagy, a particular type of autophagy, is involved in ER degradation and ER homeostasis. It is reported that different mammalian flavors of ER-phagy are mediated by different receptors. ER stress-induced receptor CCPG1 mediated ER-phagy requires ATG5, LC3 and FIP200. SEC62-mediated recovER-phagy requires ATG5, ATG7, and LC3. Most misfolded microbial-induced ER-phagy requires ATG7, ATG14, ATG16L1, BCN1 and FIP200. ER-phagy maintain the normal ER homeostasis and overall cell health through the degradation of ER membranes, removal of ER luminal protein aggregates [49, 50].

ATG7 is involved in two ubiquitin-like protein (Ubl) conjugation systems, the Atg12 conjugation system and the Atg8 conjugation system, as one of components during autophagy [51, 52]. Atg5 is critical for autophagy at the stage of autophagosome precursor synthesis. They participate in the initiation of autophagosome formation and ER-phagy process. However, whether and how their regulation of autophagy is associated with ER stress and apoptosis remained unknown. This study can clarify the relationship between autophagy, ER stress and apoptosis, as well as the molecular events in this ER-phagy process, which have important pathophysiological consequences.

Many studies have shown that both autophagy and UPR are activated under ER stress, and promote cell survival. As the UPR induces not only cell survival but also cell death signals, the well-orchestrated processes between autophagy and UPR are involved in either restoring cellular homeostasis or committing to cell death [53, 54]. We detected the expressions of autophagy related proteins increased in Tm-induced ER stress, and the expression of ER stress associated proteins, PERK、p-PERK and Nrf2, decreased dramatically in RAPA-stimulated autophagy. Interestingly, we found the expression of autophagy proteins were partially inhibited after silencing of PERK, Nrf2 or ATF4 via siRNA approach. These results suggest that ER stress interplays with autophagy in human chondrocyte (Fig. 1).

Autophagy, as a well-defined and self-digestion process for degrading proteins and organelles in response to cellular stress, can maintain the cell’s homeostasis and conducive to cell survival. More than 30 autophagy-related (ATG) genes control autophagy activation. ATG5 and ATG7, as autophagy proteins, can participate in the autophagosome formation [55, 56]. To define the interaction between autophagy, ER stress, and apoptosis, we generated adenovirus carrying ATG5 and ATG7, then infected the C28I2 cells. The result showed that overexpression of ATG5 or ATG7 can improve autophagy, increase autophagy flux and autophosome formation. Meanwhile, the ER stress was inhibited after above mentioned cells being infected with Ad-ATG5 or Ad-ATG7,as assayed by PERK, p-PERK and Nrf2 expression. Additionally, the combined effect of ATG5 and ATG7 can evidently enhance autophagy and inhibit ER stress comparing with individual treatment (Figs. 2, 3, 4).

Autophagy and apoptosis determine a cell’s fate through regulating the turnover of proteins and organelles. Normally cytoprotective function of autophagy inhibits the apoptosis induction, and serves to cell survival through suppressing apoptosis. Most of apoptosis-associated caspase activation shuts off the autophagic process and block autophagy. ATG7, an E1-like enzyme, is required to inhibit translocation of caspase-9 to the apoptosome, hence preventing apoptosis [57, 58]. ATG5 can also participate in the protective effect. The level of active caspase-3, 7 and RAPA degradation in CsA-treated cells increased after autophagy effectors ULK1, ATG5 or ATG7 were silenced [59–61].

Our data indicated that overexpression of ATG5, ATG7 and ATG5 + ATG7 can clearly reduce the apoptosis of chondrocyte comparing with the control’s. And the joint infection of them can enhance the inhibition effect. Moreover, overexpression of them can also influence cell cycle distribution. The application of ATG5, ATG7 and ATG5 + ATG7 can promote cell proliferation in chondrocyte with the G1 phase cells reduction, and the S phase cells enhancement. The joint application of ATG5 and ATG7 can promote the increasing effect of the S phase cells. These data showed that overexpression of ATG5, ATG7 and ATG5 + ATG7 can obviously inhibit apoptosis and improve cell proliferation in chondrocytes (Figs. 5).

All is known that chondrocyte apoptosis is the main cause of osteoarthritis. When cartilage destruction and matrix degradation failed to be eliminated and remain in cartilage, they can induce the chondrocyte apoptosis. Autophagy induction might be a beneficial method to relieve chondrocyte apoptosis and protect cartilage from destruction [62, 63]. Herein, we tested that overexpression of ATG5 and ATG7 can promote cell proliferation and inhibit apoptosis when they activate autophagy. There have been recent reviews on the bidirectional interaction between autophagy and ER stress. The degree of interaction between autophagy and UPR activation can determine the balance between prosurvival and antisurvival signals, in which autophagy is involved in either promote or attenuate ERS and UPR signals. We detected that both ATG5 and ATG7 overexpression can block UPR as soon as autophagy activation. It is demonstrated that both ATG5 and ATG7 can stimulate autophagy, which leads to protective effect and serves as a prosurvival mechanism through inhibition ER stress conditions (Fig. 6a, b, c, d).

Eukaryotic cell growth and proliferation are controlled by cell cycle, in which cell cycle arrest is often accompanied by autophagy induction, a crucial survival procedure during stress conditions. The above studies suggest that ATG5 and ATG7 increase the cell proliferation and inhibit ER stress and apoptosis in chondrocyte, however, this enhancement effect of ATG5 and ATG7 vanished after the cells being infected with Ad-siPERK, Ad-siNrf2 and Ad-siPERK+Ad-siNrf2 as evidenced by G1 phase arresting, S phase reducing and G2-M phase delaying (Fig. 6e, f, g, h). More importantly, the protective effect of ATG5/7 overexpression on chondrocyte survival is dependent upon PERK signaling. Interestingly, the effect of siPERK and siNrf2 on the cytoprotective effect of ATG5/7 are of synergism, while the effect of siPERK and siATF4 are of antagonism (Fig. 7). The effects of ATG5 + ATG7 on ER stress repression, autophagy enhancement, apoptosis inhibition and cell growth augment declined after treatment with siPERK and GSK2606414, a typical PERK pathway inhibitor (Fig. 8). It is indicated that ATG5 and ATG7 regulate on autophagy, ER stress, apoptosis and cell cycle through PERK signalling pathway, a vital branch UPR pathways. As reported, ATG5 and ATG7 are involved in autophosome formation and fusion of autophagosomes and lysosomes subsequently through ER-phagy, which contains a variety of processes that are both mechanistically different and regulate the delivery of ER fragments or their luminal content within lysosomes. The ER-phagy process modulates ER turnover, ER size, and clearance of ER subdomains containing proteins and lipids that are faulty or present in excess. PERK was reported to up-regulate the transcription of numerous autophagy genes and cargo receptors through ATF4 and CHOP, resulting in an augment in autophagic flux [50, 64, 65]. Our results showed that the protective effect of ATG5 and ATG7 overexpression on chondrocyte survival is dependent upon PERK signaling through ER-phagy process, suggesting that PERK signal is the pivot role for ER-phagy in normal ER homeostasis and overall cell health.

More recently, autophagy can be regulated by cell cycle control, and has been shown to engage in a complex interplay with ER stress and apoptosis. In cellular procedure, autophagy, ER-phagy and UPR act as a cell survival pathway to suppress apoptosis, and on the other hand, it can result in cell death.The molecular regulators of every pathway are interconnected and affect each other. The cross-talk among autophagy, ER stress, apoptosis and cell growth are quite intricate. All of them regulate the overall fate of the cell synergistically.

## Conclusions

**Fig. 9:**
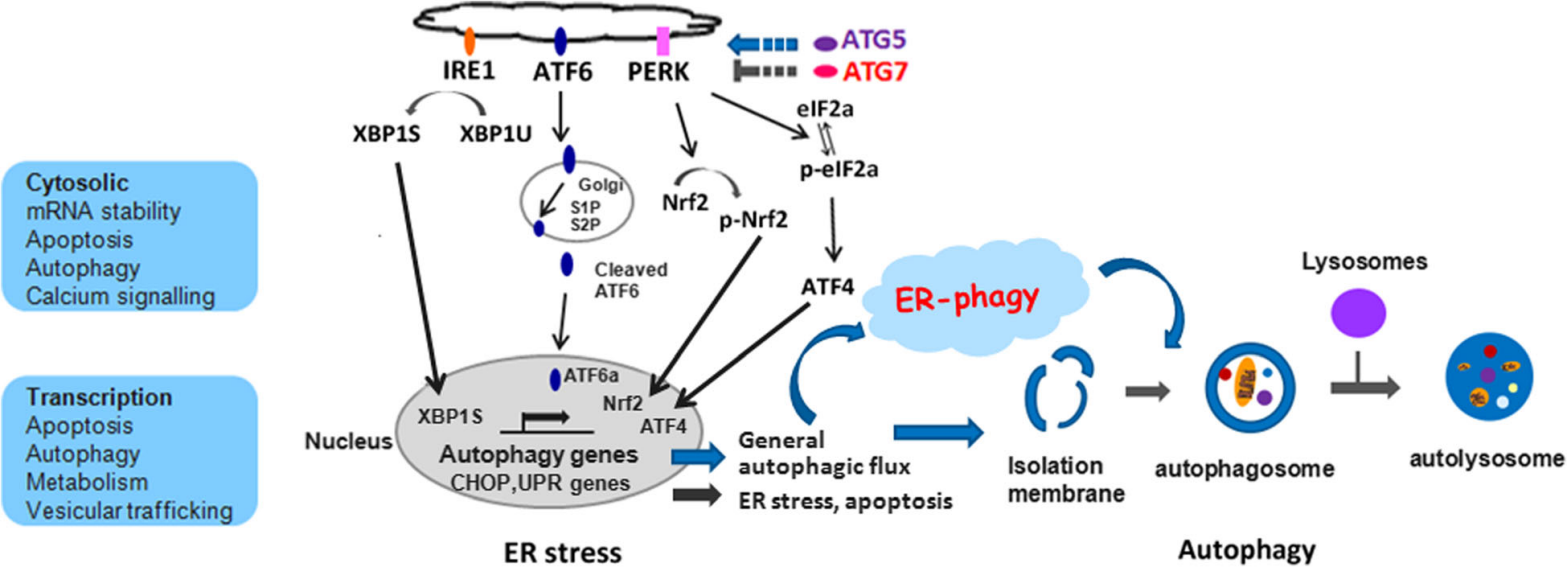
A prospective model for clarifying the role and regulation of ATG5 and ATG7 on autophagy and ER stress. ATG5 and ATG7 induce autophagy, whereas repress ER stress and apoptosis through PERK signaling, one vital signal pathway of ER stress. “→” and “⊣” indicate activation and repression, respectively

Our study provides a novel insight into the role of ATG5 and ATG7 in regulating autophagy, ER stress, ER-phagy, apoptosis and cell proliferation. As summarized in Fig. 9, we propose a model for the different role of ATG5 and ATG7 in autophagy and ER stress. ATG5 and ATG7 induce autophagy, autophagy flux and autophagosome formation, whereas inhibit ER stress in the process of cell survival and cell death through PERK signaling, one vital signal pathway of ER stress. Continued research of these and other means of crosstalk between ER stress, apoptosis and autophagy is necessary to elucidate the mechanisms controlling the balance between survival and death both under normal and disease conditions. New insight into the mechanism of autophagy affect ER stress responses will open new approach to the involvement of ER-phagy in ER homeostasis and the development of molecular target-based treatment of cartilage disorders and osteoarthritis.

## Additional file

Additional file 1: The results of DNA sequencing and the adenoviral vector endonuclease identification. (PDF 344 kb)

## Abbreviations

ATF6: activating transcription factor 6
ER: endoplasmic reticulum
IRE1: inositol-requiring enzyme 1
LAMP-1: lysosome-associated membrane proteins 1
LC3: Microtubule-associated protein 1α/β-light chain 3
PERK: protein kinase RNA (PKR)-like ER kinase
PI: propidium iodide
TEM: transmission electron microscope
Tg: thapsigargin
Tm: tunicamycin
UPR: unfolded protein response
