# Supplementary material for: ATG5 and ATG7 induced autophagy interplays with UPR via PERK signaling
Source: Cell Commun Signal. 2019 May 6;17:42. doi: 10.1186/s12964-019-0353-3 (PMC6503447; doi:10.1186/s12964-019-0353-3)
Supplement: Supplementary file 1 — The results of DNA sequencing and the adenoviral vector endonuclease identification. (PDF 344 kb) [file 12964_2019_353_MOESM1_ESM.pdf]

Primer sequence

ATG5-F(正向)
5’ GTCAGATCCGCTAGAGATCTGCTTACTAAGTTTGGCTTTGGTT 3’
ATG5-R(反向)
5’ GATATCTTATCTAGAAGCTTAAGGGTGACATGCTCTGATAAAT 3’
ATG7-F(正向)
5’-GTCAGATCCGCTAGAGATCTAAATAATGGCGGCAGCTACGG -3’
ATG7-R(反向)
5’-GATATCTTATCTAGAAGCTTGGGCCATCTCAGATGGTCTCATC-3’

Enzyme site: BglⅡ、HindⅢ

pAdTrack-ATG7 plasmid endonuclease digesting and DNA sequencing

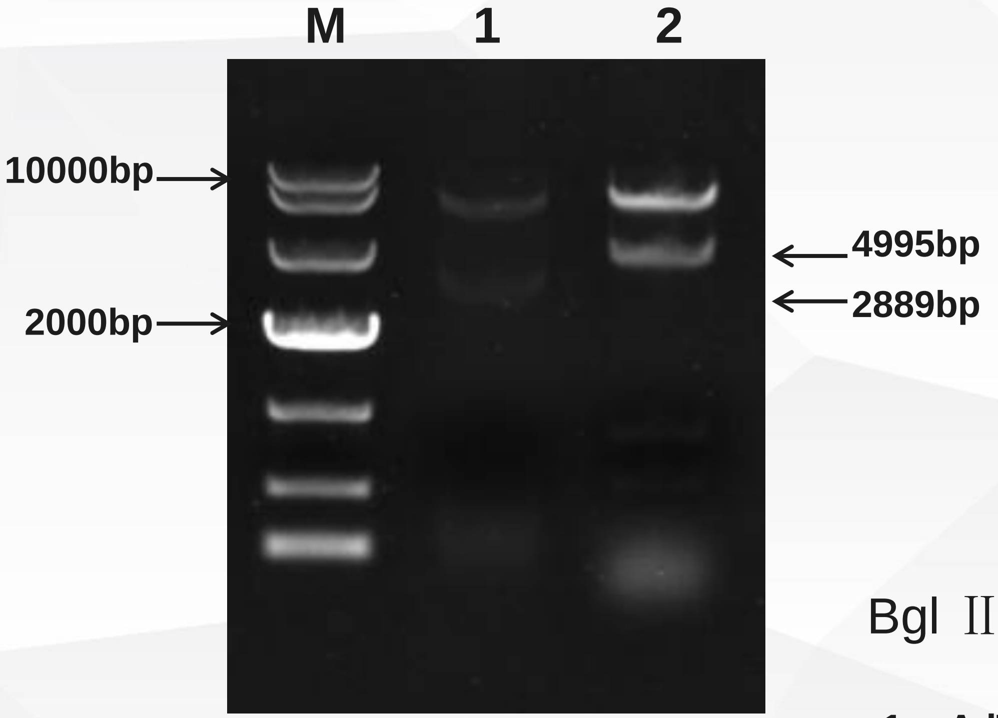

Bgl Ⅱ、Hind Ⅲ(pAdTrack-ATG7)

1: pAdTrack-CMV; 2: pAdTrack-ATG7

ATG7 sequencing

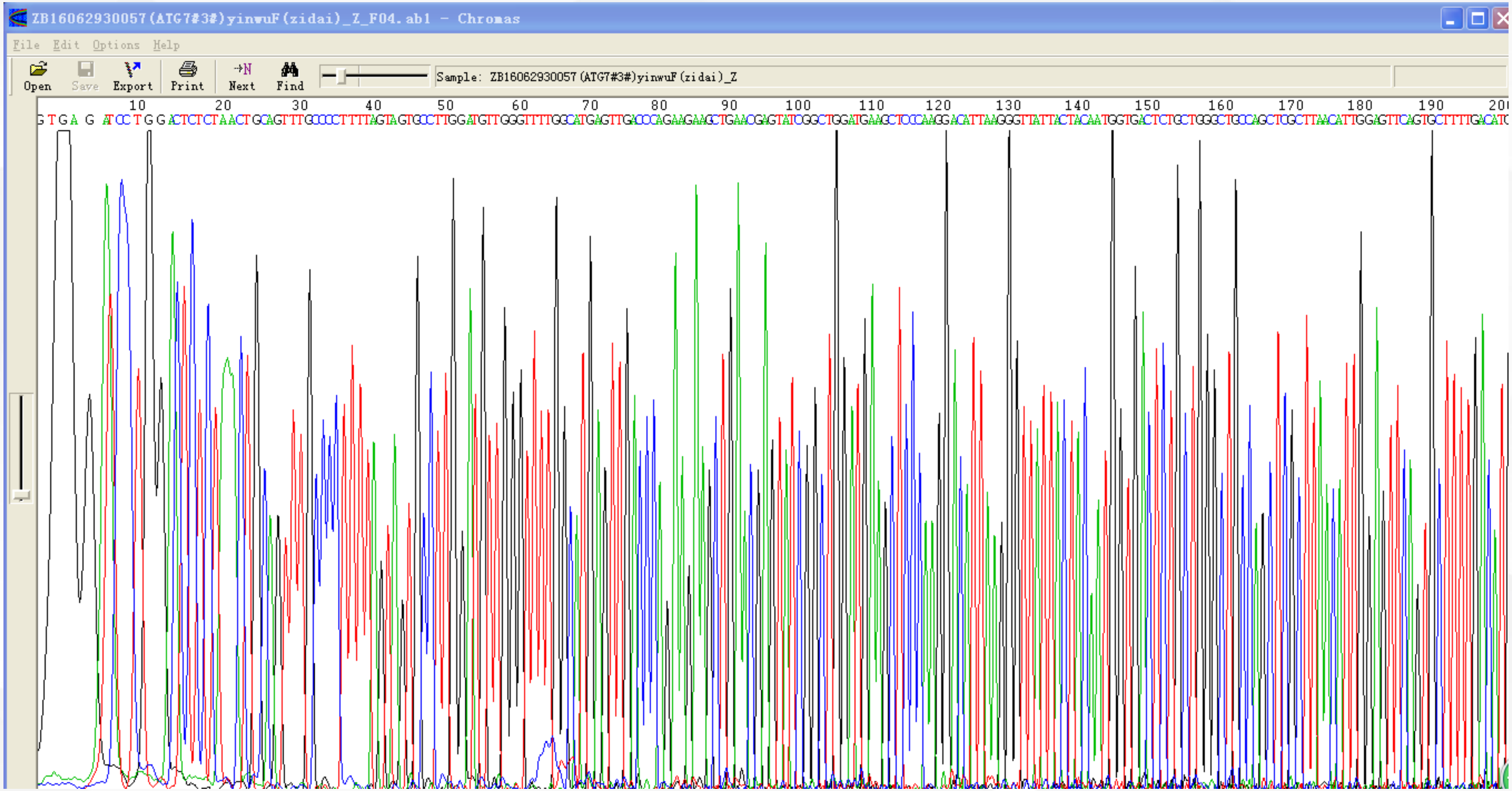

ATG7 sequencing

GTGAGATCCTGGACTCTCTAACTGCAGTTTGCCCCTTTTAGTAGTGCCTTGGATGTTGGGTTTT
GGCATGAGTTGACCCAGAAGAAGCTGAACGAGTATCGGCTGGATGAAGCTCCCAAGGACATT
AAGGGTTATTACTACAATGGTGACTCTGCTGGGCTGCCAGCTCGCTTAACATTGGAGTTCAGT
GCTTTTGACATGAGTGCTCCCACCCCAGCCCGTTGCTGCCCAGCTATTGGAACACTGTATAAC
ACCAACACACTCGAGTCTTTCAAGACTGCAGATAAGAAGCTCCTTTTGGAACAAGCAGCAAAT
GAGATATGGGAATCCATAAAATCAGGCACTGCTCTTGAAAACCCTGTACTCCTCATCAAGTTCC
TCCTCTTGACATTTGCAGATCTAAAGAAGCACCACTTCTACTATTGGTTTTGCTATCCTGCCCTC
TGTCTTCCAGAGAGTTTACCTCTCATTCAGGGGCCAGTGGGTTTGGATCAAAGGTTTTCACTA
AAACAGATTGAAGCACTAGAGTGTGCATATGATAATCTTTGTCAAACAGAAGGAGTCACAGCTC
TTCCTTACTTCTTAATCAAGTATGATGAGAACATGGTGCTGGTTTCCTTGCTTAAACACTACAGT
GATTTCTTCCAAGGTCAAAGGACGAAGATAACAATTGGTGTATATGATCCCTGTAACTTAGCCC
AGTACCCTGGATGGCCTTTGAGGAATTTTTTGGTCCTAGCAGCCCACAGATGGAGTAGCAGTT
TCCAGTCTGTTGAAGTTGTTTGCTTCCGTGACCGTACCATGCAGGGGGCGAGAGACGTTGCC
CACAGCATCATCTTCGAAGTGAAGCTTCCAGAAATGGCATTTAGCCCAGATTGTCCTAAAGCA
GTTGGGATGGGAAAAGAACCAGAAAGGAGGCATGGACCAAGATGGTGAACCTCAGTGATGTA
TGGACCCTAAAGTTAGCTGAGTCATCAGTGGATCTAATCTCAACTGATGTGTGAGATGGCTACT
TTAGACTGACAGTGTGTCTGTCAATGTCTGCTGCTGAGCGCACTGGAGCATGTAGCTAGACGT
GATGGATGGGGCGTGGACCAATCACGGTTGTGGACAATGCCCAGATTCTCTAACTCCGATCTT
GTGAAGTACCCTCTAGATTGGAATGCTAGGGTGTTGACCAAGTCCTTGGGGCA

pAdTrack-ATG5 plasmid endonuclease digesting and DNA sequencing

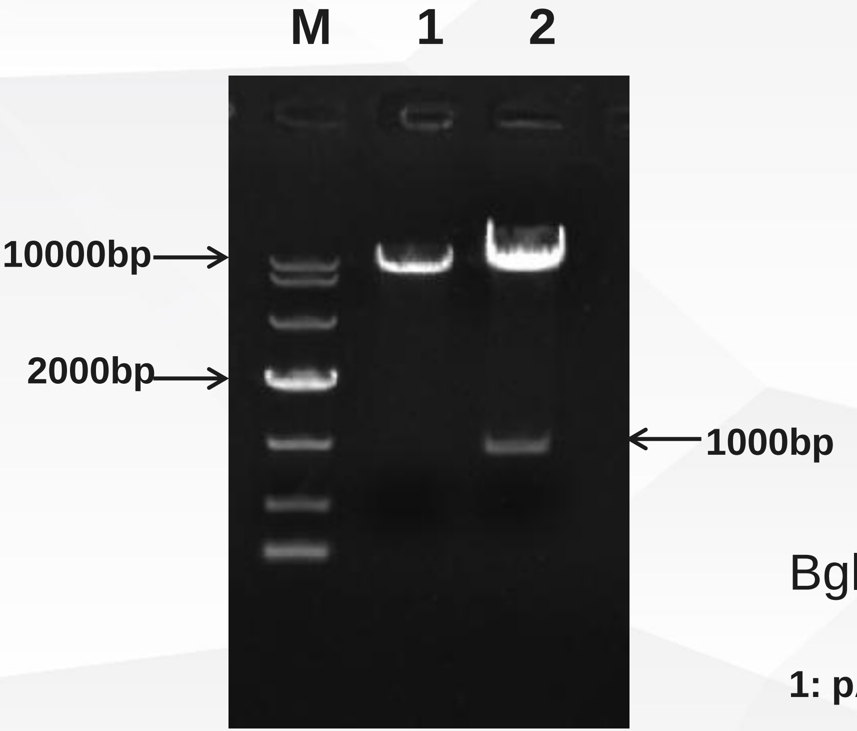

Bgl Ⅱ、Hind Ⅲ(pAdTrack-ATG5)

1: pAdTrack-CMV; 2: pAdTrack-ATG5

ATG5 sequencing

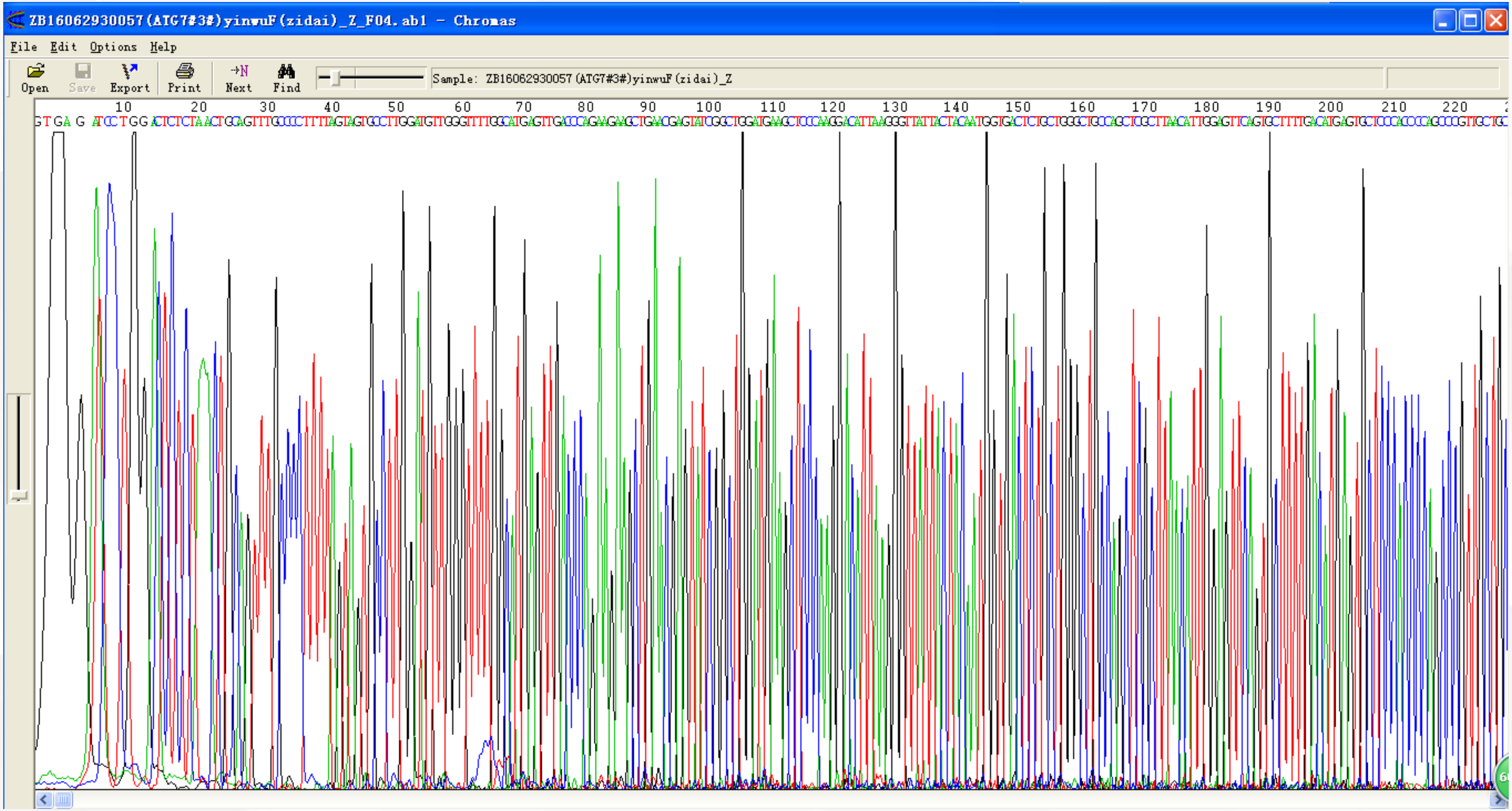

ATG5 sequencing

AAGGAATAAAGAATTTAGCCTGTATGTACTGCTTTAACTCCTGGAAGAATGACAGATGACAAAGAT
GTGCTTCGAGATGTGTGGTTTGGACGAATTCCAACTTGTTTCACGCTATATCAGGATGAGATAAC
TGAAAGGGAAGCAGAACCATACTATTTGCTTTTGCCAAGAGTAAGTTATTTGACGTTGGTAACTG
ACAAAGTGAAAAAGCACTTTCAGAAGGTTATGAGACAAGAAGACATTAGTGAGATATGGTTTGAA
TATGAAGGCACACCACTGAAATGGCATTATCCAATTGGTTTGCTATTTGATCTCCTTGCATCAAGT
TCAGCTCTTCCTTGGAACATCACAGTACATTTTAAGAGTTTTCCAGAAAAAGACCTTCTGCACTG
TCCATCTAAGGATGCAATTGAAGCTCATTTTATGTCATGTATGAAAGAAGCTGATGCTTTAAAACAT
AAAAGTCAAGTAATCAATGAAATGCGGAAAAAAGATCACAAGCAACTCTGGATGGGATTGCAAAA
TGACAGATTTGACCAGTTTTGGGCCATCAATCGGAAACTCATGGAATATCCTGCAGAAGAAAATG
GATTTCGTTATATCCCCTTTAGAATATATCAGACAACGACTGAAAGACCTTTCATTCAGAAGCTGT
TTCGTCCTGTGGCTGCAGATGGACAGTTGCACACACTAGGAGATCTCCTCAAAGAAGTTTGTCC
TTCTGCTATTGATCCTGAAGATGGGGAAAAAAAGAATCAAGTGATGATTCATGGAATTGAGCCAA
CGTTGGAAACACCTCTGCAGTGGCTGAGTGAACATCTGAGCTACCCGGATAATTTTCTTCATATT
AGTATCATCCCACAGCCAACAGATTGAAGGATCAACTATTTGCCTGAACAGAATCATCCTTAAATG
GGATTTATCAGAGCATGTCACCCTTAAGCTTCTAGATAAGATATCCGATCCACCGGATCTAGATAA
CTGATCATAATCAGCCATACCACATTTGTAGAGGTTTTACTTGCTTTAAAAACCTCCCACACCTCC
CCTGAACCTGAAACATAAATGATGCATTGTGTGTTAACTTGTTTTATTGCAGCTATATGGTACAAAT
AAAGCATAGCATCACAAATTTCACAAAATAAAGCATTTTTTTCCACTGCCATCCTAGTGTGGTTGTC
CAACTATCATGATCCTAACGCGGAGGTTTATCGACGACTGTCTAGTGATTATTAGGTTAATTC
